# Downregulated RPS-30 in *Angiostrongylus cantonensis* L5 plays a defensive role against damage due to oxidative stress

**DOI:** 10.1186/s13071-020-04495-3

**Published:** 2020-12-09

**Authors:** Wei-Wei Sun, Xiu-Mei Yan, Qing Shi, Yuan-Jiao Zhang, Jun-Ting Huang, Hui-Cong Huang, Hong-Fei Shi, Bao-Long Yan

**Affiliations:** 1 Department of Parasitology, School of Basic Medical Sciences, Wenzhou Medical University, Wenzhou, 325035 Zhejiang PR China; 2 Department of Biochemistry, School of Basic Medical Sciences, Wenzhou Medical University, Wenzhou, 325035 Zhejiang PR China; 3 Department of Pediatric Gastroenterology, The Second Affiliated Hospital and Yuying Children’s Hospital of Wenzhou Medical University, Wenzhou, 325000 Zhejiang China; 4 School of First Clinic Medicine, Wenzhou Medical University, Wenzhou, 325035 Zhejiang PR China; 5 Henan Provincial Engineering Laboratory of Insects Bio-reactor, China-UK-NYNU-RRes Joint Laboratory of Insect Biology, Nanyang Normal University, Nanyang, 473061 PR China

**Keywords:** *Angiostrongylus cantonensis*, RPS-30, *Caenorhabditis elegans*, *Oxidative stress*, *Apoptosis*

## Abstract

**Background:**

Eosinophilic meningitis, caused by fifth-stage larvae of the nematode (roundworm)* Angiostrongylus cantonensis*, is mainly attributed to the contribution of eosinophils to tissue inflammatory responses in helminthic infections. Eosinophils are associated with the killing of helminths via peroxidative oxidation and hydrogen peroxide generated by the dismutation of superoxide produced during respiratory bursts. In contrast, when residing in the host with high level of eosinophils, helminthic worms have evolved to attenuate eosinophil-mediated tissue inflammatory responses for their survival in the hosts. In a previous study we demonstrated that the expression of the *A. cantonensis* RPS 30 gene (*Acan*-*rps-*30) was significantly downregulated in *A. cantonensis* L5 roundworms residing in cerebrospinal fluid with a high level of eosinophils. *Acan*-RPS-30 is a protein homologous to the human Fau protein that plays a pro-apoptotic regulatory role and may function in protecting worms from oxidative stress.

**Methods:**

The isolation and structural characterization of *Acan*-RPS-30 were performed using rapid amplification of cDNA ends (RACE), genome walking and bioinformatics. Quantitative real-time-PCR and microinjection were used to detect the expression patterns of *Acan-rps-*30. Feeding RNA interference (RNAi) was used to knockdown the apoptosis gene* ced-3*. Microinjection was performed to construct transgenic worms. An oxidative stress assay was used to determine the functions of *Acan*-RPS-30.

**Results:**

Our results showed that *Acan-*RPS-30 consisted of 130 amino acids. It was grouped into clade V with *C. elegans* in the phylogenetic analysis. It was expressed ubiquitously in worms and was downregulated in both L5 larvae and adult *A. cantonensis*. Worms expressing *pCe-rps*30*::Acan-rps-*30*::rfp*, with the refractile “button-like” apoptotic corpses, were susceptible to oxidative stress. Apoptosis genes *ced-*3 and *ced-*4 were both upregulated in the transgenic worms. The phenotype susceptible to oxidative stress could be converted with a *ced-*3 defective mutation and RNAi. *rps-*30^−/−^ mutant worms were resistant to oxidative stress, with *ced-*3 and *ced-*4 both downregulated. The oxidative stress-resistant phenotype could be rescued and inhibited by through the expression of *pCe-rps*30*::Acan-rps-*30*::rfp* in *rps-*3^−/−^ mutant worms.

**Conclusion:**

In *C. elegans* worms, downregulated RPS-30 plays a defensive role against damage due to oxidative stress, facilitating worm survival by regulating downregulated *ced-3*. This observation may indicate the mechanism by which *A. cantonensis* L5 worms, with downregulated *Acan-*RPS-30, survive in the central nervous system of humans from the immune response of eosinophils.

**Graphic abstract:**

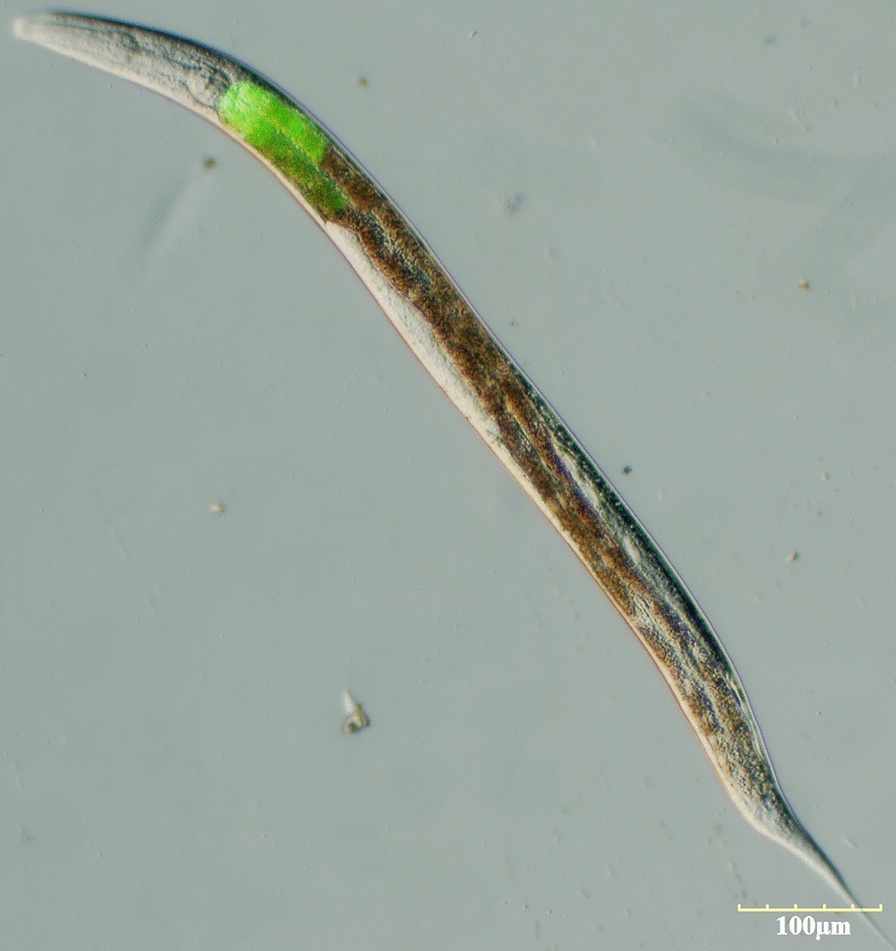

## Background

*Angiostrongylus cantonensis* is a human zoonotic pathogen that may cause eosinophilic meningitis [1]. Several different hosts are required to complete the life-cycle of *A. cantonensis*. Humans are an atypical host are are mainly be infected through accidental ingestion of undercooked intermediate hosts, such as the golden apple/channeled apple snail *Pomacea canaliculata* in which the infective third-stage larvae (iL3) resides [2]. After passage to the small intestine, iL3 will penetrate the blood–brain barrier, subsequently infecting the central nervous system where it will develop into the fifth-stage larvae (L5) and cause angiostrongyliasis with neurological symptoms [3–6].

Eosinophils, recruited from the circulation into the central nervous system [3], are robust producers of extracellular superoxide due to expression of high levels of the enzyme complex that generates superoxide [7], thereby contributing to tissue inflammatory responses and host defense in helminthic infections [8]. Eosinophil peroxidase (EPO), resident in the granule matrix of eosinophils, would be released in this response [9, 10]. EPO is associated with the killing of helminths through peroxidative oxidation and the hydrogen peroxide (H_2_O_2_) generated by dismutation of the superoxide produced during respiratory bursts [11–13]. In contrast, residing in a host with high levels of eosinophils, helminthic worms have evolved to attenuate eosinophil-mediated tissue inflammatory responses to facilitate their survival in that host [8]. Therefore, *A. cantonensis* L5 that reside in the cerebrospinal fluid together with eosinophils may be resistant to damage by oxidative stress. In a previous study, we showed that the expression level of *A. cantonensis* ribosomal protein 30 (*Acan*-RPS-30) was lower in L5 than in iL3, based on the proteomic analysis of different developmental stages using two-dimensional difference gel electrophoresis [4].

*Acan-rps-*30 is a homologous gene of human *Fau* [14] (FBR-MuSV associated ubiquitously expressed gene), which was originally isolated from a radiation-induced osteosarcoma [15]. *Fau* is inversely inserted as the fox sequence in FBR-MuSV [16, 17], and expression of fox enhances the transformation of FBR-MuSV, presumably by inactivating *Fau* expression [18, 19]. *Fau* may play an important role in inhibiting tumorigenesis, based on results showing that it is downregulated in both breast cancer [20] and ovarian cancer [21].* Fau* also regulates apoptosis in human T-cell lines and HEK293/17 cells [20]. A sequence antisense to *Fau* is able to decrease apoptosis induced by dexamethasone, ultraviolet light or cisplatin in W7.2c cells [19]. In the parasitic nematode *Haemonchus contortus*, RPS-30 can regulate the fourth-stage larval diapauses [22]. *Fau* encodes an ubiquitin-like protein (UBiL) fused to ribosomal protein S30 (S30) as a carboxy-terminal extension [14]. These two products are thought to result from post-translational cleavage [23]. Human Fau-UBiL has 37% amino acid sequence similarity to ubiqutin and contains the C-terminal Gly-Gly dipeptide motif that participates in isopeptide bond formation between the ubiquitin and lysine of target proteins [14]. However, a lack of internal lysine residues, which are sites of poly-ubiquitin chain formation, indicates that the biological function of UBiL is different from that of ubiquitin [23]. The identification of UBiL covalently bound to Bcl-G, a member of the Bcl-2 family of apoptosis control proteins [24], suggests a pro-apoptotic regulatory role for *Fau*, mediated via Bcl-G [19, 23].

Apoptosis is closely related to oxidative stress in many cell lines, both mammalian and the model organism *Caenorhabditis elegans* [25, 26]. Therefore, in this study, our aim was to determine the structures and functions of *Acan-*RPS-30 in *A. cantonensis* L5 in order to investigate its role in regulating oxidative stress resistance.

## Methods

### Propagation of *A. cantonensis* and *C. elegans*

*Angiostrongylus cantonensis* ZJ strain was maintained and propagated in Wenzhou Medical University, China by cycling through Sprague-Dawley (SD) rats fed *Pomacea canaliculata*, as described previously [4]. The intermediate host *P. canaliculata* were infected with *A. cantonensis* L1 through feeding on rat feces. L3 were collected at 20 days post-infection. Infected snails were shelled and crushed; the intestines and other organs were then removed and the remaining tissue homogenized. The homogenates were filtered through a 40-mesh sieve, left to stand for 5 min at 4 ℃ and then precipitated 2–3 times at room temperature. The sediments were removed, and the number and viability of L3 were determined by direct observation under a light microscope. Three-week-old SD rats (weight 100–120 g, grade clean, certificate SYXK [ZHE] 2005-0061), supplied by the Laboratory Animal Center of Wenzhou Medical University, were orally infected with 50 L3 per rat. The rats were housed in polypropylene cages with free access to food and water and then sacrificed by anesthesia at 25 days and 45 days post-infection, respectively. The L3 worms were collected from the intermediate hosts *P. canaliculata*; the L5 were harvested from the brains of mice (C57BL/6J [B6], Certificate SYXK [zhe2015-0009]) (non-permissive host same as humans), which were orally infected with 30 L3 per mouse; the adult worms were collected from the blood vessels of the hearts and lungs. Individuals of different sexes were separated using morphological criteria: females are usually longer and thinner than males, and males exhibit typical copulatory bursa. L3, L5 and adults were washed three times with 0.01 mol/l phosphate buffered saline and stored at − 80 °C. These rats were not used for any other part of the study.

*Caenorhabditis elegans* strains N2, *rps-30* (*tm6034/nt1*) and *ced-3* (*ok2734*) were maintained on Nematode Growth Media agar plates at 15 °C, as described previously [27]. Worms were fed *Escherichia coli* strain OP50 unless otherwise stated. The mutant strain *ced-3* (*ok2734*) was obtained from the* Caenorhabditis* Genetic Center (CGC) of the University of Minnesota (Minneapolis, MN, USA). The mutant strain *rps-30* (*tm6034/nt1*) was originally provided by Shohei Mitani of Tokyo Women’s Medical University School of Medicine (Tokyo, Japan). The gene *rsp-*30 is essential for the survival of the worms, and if the gene is deleted, worms are sterile. Therefore, the mutant strain *tm6034/nt1* was used to produce trans-heterozygous animals using a translocational balancer (nT1) that has fluorescent marker: fluorescence-positive animals carry nT1 but animals without nT1 are considered to be homozygous for the mutation.

### Isolation, purification, treatment and storage of nucleic acids

Total genomic DNA was extracted from *A. cantonensis* ZJ strain adult worms using a small-scale genomic DNA extraction Kit (Takara Biotechnology Co. Ltd., Kusatsu, Shiga, Japan). Total RNA was extracted from worms at different developmental stages employing TRIzol reagent (Invitrogen, Carlsbad, CA, USA), followed by treatment with 2 U of DNase I (Takara Biotechnology Co., Ltd.). First-strand cDNA was obtained using the M-MLV RTase cDNA Synthesis kit (Takara Biotechnology Co., Ltd.). Both DNA and RNA samples were stored at − 80 °C until used.

### Isolation of full-length cDNA and genomic DNA encoding *Acan-rps-30* from *A. cantonensis*

Using two degenerate primers, rps-30DF and rps-30DR (Additional file 1: Table S1), designed on the basis of a relatively conserved S30 domain, with reference to the *C. elegans* gene (NC_003283.11) and *Homo sapiens* gene (NC_000011.10), a portion of *Acan-rps-*30 was amplified by PCR from cDNA synthesized from total RNA extracted from adult worms. PCR products were cloned into the pMD18-T vector (Takara Biotechnology Co., Ltd.) and sequenced. Based on the available sequence information, gene-specific primer pairs (Additional file 1: Table S1) were then designed. Using 5′- and 3′- rapid amplification of cDNA ends (RACE) method (Takara Biotechnology Co., Ltd.), we obtained two partially overlapping cDNA fragments. The products were cloned into the pMD18-T vector and sequenced. Based on these sequences, we designed additional primers (Additional file 1: Table S1) to amplify the full-length *Acan-rps-30*.

Full-length genomic DNA of *Acan-rps-30* from the ZJ strain of *A. cantonensis* was obtained using a Genome Walking kit (Takara Biotechnology Co., Ltd.), using primers designed based on the acquired cDNA sequence (Additional file 1: Table S1), following the manufacturer’s instructions. The third-round PCR products were cloned into a pMD18-T vector and sequenced.

### Bioinformatics analysis

A sequence alignment between *Acan-*RPS-30, *Hs*-RPS-30 (NP_001988.1) and *Ce*-RPS-30 (NP_505007.1) was generated using Clustal Omega. Homology models were built by SWISS-MODEL using *H. sapiens* ribosome (Protein Data Bank codes 5LKS and 2L7R) as templates. Three-dimensional structural analysis was performed using the PyMOL program. All calculations were carried out under default conditions.

The amino acid sequence inferred for *Acan-*RPS-30 and seven other selected homologous sequences were subjected to phylogenetic analyses. The phylogenetic analysis was conducted using the neighbor-joining (NJ) and maximum parsimony (MP) methods, respectively, based on the Jones-Taylor-Thornton (JTT) model [28]. Confidence limits were assessed using a bootstrap procedure with 1000 pseudo-replicates for NJ and MP trees, and other settings were obtained using the default values in MEGA v.5.0. A 50% cut-off value was implemented for the consensus tree.

### Quantitative real-time PCR analysis

Quantitative real-time PCR (qRT-PCR) was performed to determine the abundance of *Acan-rps-30* transcripts in different developmental stages (L3, L5 female, L5 male, adult female, adult male) of *A. cantonensis*.

Gene expression levels were determined by RT-PCR using the SYBR®Green PCR Master Mix and a 7500 Real-Time PCR System (Applied Biosystems, Foster City, CA, USA). Relative gene expression was compared with the 18S ribosomal RNA gene (GenBank: AY295804) as an internal loading control. The target genes and the primers used are listed in Additional file 1: Table S1. Statistical analysis was conducted using a one-way analysis of variance, with* P* < 0.05 set as the criterion for significance.

### RNA interference feeding experiments

To generate *ced-3*-specific RNA interference (RNAi) vectors, *ced-3* cDNAs was cloned into the the *L4440* vector. Plasmids were transformed into *E. coli* strain HT115*.* Primers used for PCR analysis are listed in Additional file 1: Table S1. RNAi plates and media were prepared according to Kwon et al. [29]. Gravid adults of *C. elegans* were allowed to lay eggs overnight on the RNAi plates and adult worms were picked off. *Escherichia coli* containing the empty vector were used on separate plates as negative controls.

### Transgenic worms

**Fig. 1:**
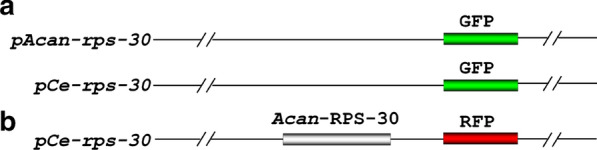
Cloning strategy for the reporter and rescuing constructs. **a** Sequences upstream of* Acan-rps*-30 5′-UTRs, about 2000 bp, was used as putative promoters. The promoter regions of *Acan-rps-*30 and *Ce-rps-*30, fused with green fluorescent protein (*GFP*) downstream, were cloned into plasmid pPD95.77 to construct *pAcan-rps-*30*::gfp* and *pCe-rps*30*::gfp*, respectively. **b**The *Acan-rps-*30 cDNA sequence, fused with red fluorescent protein (*RFP*) downstream was cloned into plasmid pPD95.77, using *pCe-rps*30 as promoter, to construct *pCe-rps-*30*::Acan-rps-*30*::rfp*.* Acan-RPS-30* *Angiostrongylus cantonensis* ribosomal protein 30, *UTR* Untranslated region

A sequence upstream of *Acan-rps-30* 5′-UTRs, approximately 2000 bp, was used as the putative promoter. To analyze promoter activity of *Acan-rps-30*, the promoter regions of *Acan-rps-30* and *Ce-rps-30* were amplified and cloned into plasmid pPD95.77 to construct *pAcan-rps-30::gfp* and *pCe-rps30::gfp*, respectively (Fig. 1a).

To perform cross-species expression of *Acan-*RPS-30 in the N2 strain and *rps-30* (*tm6034/nt1*) strains, the cDNA sequence was amplified and cloned into pPD95.77 using the promoter of *Ce-rps30* to construct plasmid *pCe-rps-30::Acan-rps-30::rfp* (Fig. 1b). All primers used are listed in Additional file 1: Table S1.

Recombinant plasmids were each microinjected into the gonad of young, adult *C. elegans* hermaphrodites as described previously [2, 30], together with plasmid pRF4 containing a dominant mutant allele of the *rol-6* gene, each at a final concentration of 50 μg/ml in the same mixture, using the pPD95.77 (*pCe-rps30::gfp*) and pRF4 plasmid mixture as a control. The F2 and subsequent generations with a roller phenotype were analyzed and selected to examine the expression patterns of green fluorescent protein (GFP) or red fluorescent protein (RFP), using a fluorescent microscope (Olympus model IX71; Olympus Corp., Tokyo, Japan). A minimum of three independent lines expressing each transgene were evaluated*.*

### Oxidative stress assay

The oxidative stress assay was performed as described previously [31]. Briefly, adult hermaphrodites (30 worms/group) were transferred to a 96-well plate containing M9 buffer with 3 mM H_2_O_2_. After incubation at 20 °C for the specified durations, the number of dead worms was determined. Worms were scored as dead when they no longer responded with movement to light prodding of the head. Three (H_2_O_2_) independent experiments were performed. Statistical analysis was performed with Microsoft Excel 2010 software (Microsoft Corp., Redmond, WA, USA) using an unpaired two-tailed t-test.

## Results

### Structural characterisation of* Acan*-RPS-30

**Fig. 2:**
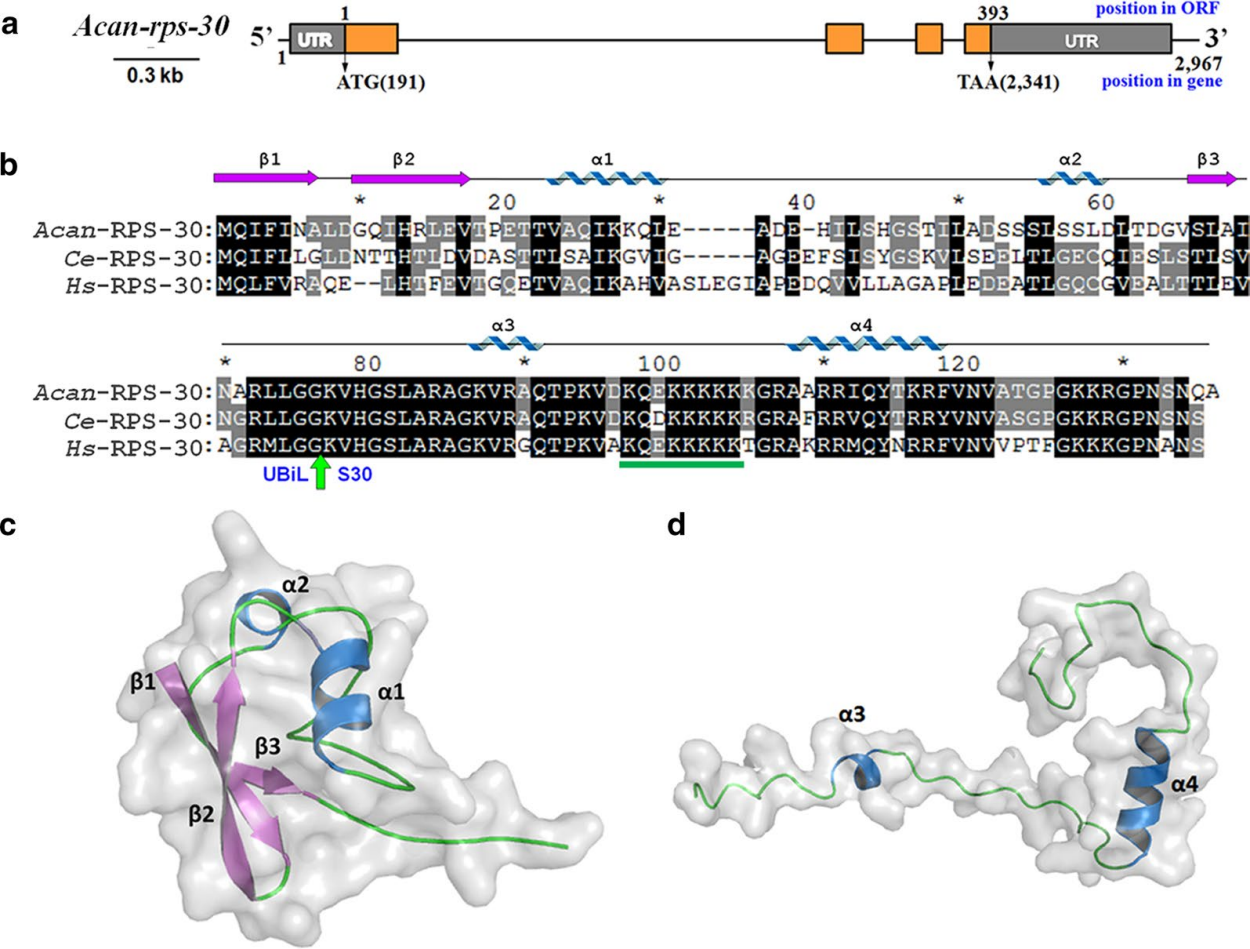
Structure and sequence analysis of *Acan-*RPS-S30. **a** The exon–intron organization of *Acan-rps-*30. The *Acan-rps-*30 gene, from *A. cantonensis*, spans 2967 bp and consists of 4 exons and 3 introns. The narrow bar represents untranscribed sequences or introns; the wide bars represent exons; brown blocks are coding regions; gray blocks are the non-coding 5′- and 3′-UTR.* ORF* Open reading frame. **b** Alignment of amino acid sequences of *Acan-*RPS-S30 with those from* Homo sapiens* (*Hs*-RPS-30) and *Caenorhabditis elegans* (*Ce*-RPS-30). The accession numbers of sequences available from current databases are: NP_505007.1 (*Ce*-RPS-S30) and NP_001988.1 (*Hs*-RPS-30). Identical and similar residues are shown in black and gray blocks, respectively. The potential cleavage sites (Gly-Gly) of the fusion protein (ubiquitin-like [*UBiL*]-ribosome protein S30 [S30]) are indicated with green arrow (upstream and downstream sequences are UBiL and S30 regions, respectively). The nuclear location signals in the S30 regions are indicated by a green line underneath the sequences. The secondary structural elements of *Acan-*RPS-S30 are shown above the alignment. **c** Predicted tertiary structure of UBiL region, showing 3 β-sheets and 2 α-helixes. **d** Predicted tertiary structure of S30 region, showing 2 α-helixes

The complete cDNAs of *Acan-rps-*30 was isolated by RACE from *A. cantonensis*. *Acan-rps-*30 cDNA was 1209 bp in length, including an open reading frame (ORF) of 393 bp (including stop codon), a 5’-untranslated region (UTR) of 190 bp, and a 3′-UTR of 626 bp (Fig. 2a). The 5′-UTR harbored the consecutive pyrimidines (TTTCTTTTC), which are commonly found at the 5′ end of eukaryotic ribosomal protein mRNAs [17] and which may play a role in regulating translation [32]. The 3′-UTR contained the hexamer AATAAA (positions, 612 bp downstream of the TAA). The complete *Acan-rps-*30 gene, isolated by Genome Walking from genomic DNA of *A. cantonensis*, was 2967 bp in length, consisting of four exons and three introns (Fig. 2a).

To characterize the structure of *Acan-*RPS-S30, sequence alignment and structural analysis were performed. The cDNA of *Acan-rps-*30 encoded predicted proteins of 130 amino acids (Fig. 2b), which contained the potential cleavage sites (Gly-Gly) of the fusion protein (UBiL-ribosome protein S30 [S30]). The amino acids sequence was aligned with those from* Homo sapiens* (*Hs*-RPS-30) and *Caenorhabditis elegans* (*Ce*-RPS-30) (Fig. 2b). The results showed that the C-terminal S30 domains were conserved (*Acan*-RPS-S30* vs* *Ce*-RPS-S30 and *Hs*-RPS-30, with 87.9 and 77.6% similarity, respectively), whereas the N-terminal UBiL domains were divergent (37.5 and 30.4% similarity, respectively). The S30 domain contained a nuclear location signal (NLS), KQEKKKKKK, with which RPS-30 can go into the nucleus and involve itself in the small subunit assembly of ribosome. Structural analysis from homology models revealed that the UBiL region possessed three β-sheets and two α-helixes (Fig. 2c), and the S30 region contained two α-helixes (Fig. 2d). The UBiL region did not harbor the K48 and K63 residues, sites of poly-ubiquitin chain formation, consistent with the orthologues from other species, indicating different functions [23], although the structure of UBiL was similar to that of ubiquitin.

### Evolutionary relationship of *Acan*-RPS-30 with RPS-30 orthologues from other nematode species

**Fig. 3:**
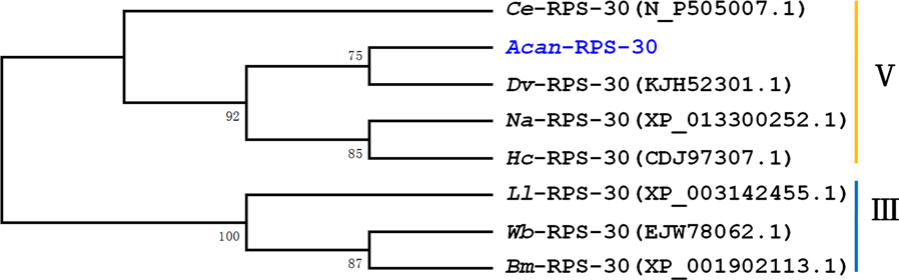
Neighbor-joining phylogenetic tree of RPS-30 proteins from several nematodes. The tree is calculated using the Jones-Taylor-Thornton model in the MEGA program version 5.0. Bootstrap values above the branches (1000 iterations) are shown for robust clades (> 70%).* Ce* *Caenorhabditis elegans*,* Acan* *A. cantonensis*,* Dv* *Dictyocaulus viviparous*,* Na* *Necator americanus*,* Hc* *Haemonchus contortus*,* Ll* *Loa loa*,* Wb* *Wuchereria bancrofti Bm* *Brugia malayi*. The corresponding accession numbers are listed on the right of each species. Clade numbers are given in Roman numerals

To determine the evolutionary relationship between *A. cantonensis* and other nematodes, the predicted amino acid sequence of *Acan*-RPS-30 was aligned with orthologues from other nematodes and subjected to phylogenetic analyses (Fig. 3). *Acan*-RPS-30 clustered closely with *Dv*-RPS-30 from *Dictyocaulus viviparus*, with a similarity of 89.2%. Cladistic analysis showed that the RPS-30 homologues selected from seven parasitic nematodes were mainly grouped into two clades. *Haemonchus contortus*, *Necator americanus*, *D. viviparus* and *A. cantonensis* were in clade V; *Wuchereria bancrofti*, *Brugia malayi* and *Loa loa* were in clade III. This result is in agreement with a modern phylogenetic analysis of nematodes [33]. When sequences from the S30 regions only were analyzed, bootstrapping did not support the clusters (data not shown), possibly indicating that the divergences of the UBiL regions are likely related to species specificity.

### The expression patterns of *Acan*-rps-30

**Fig. 4:**
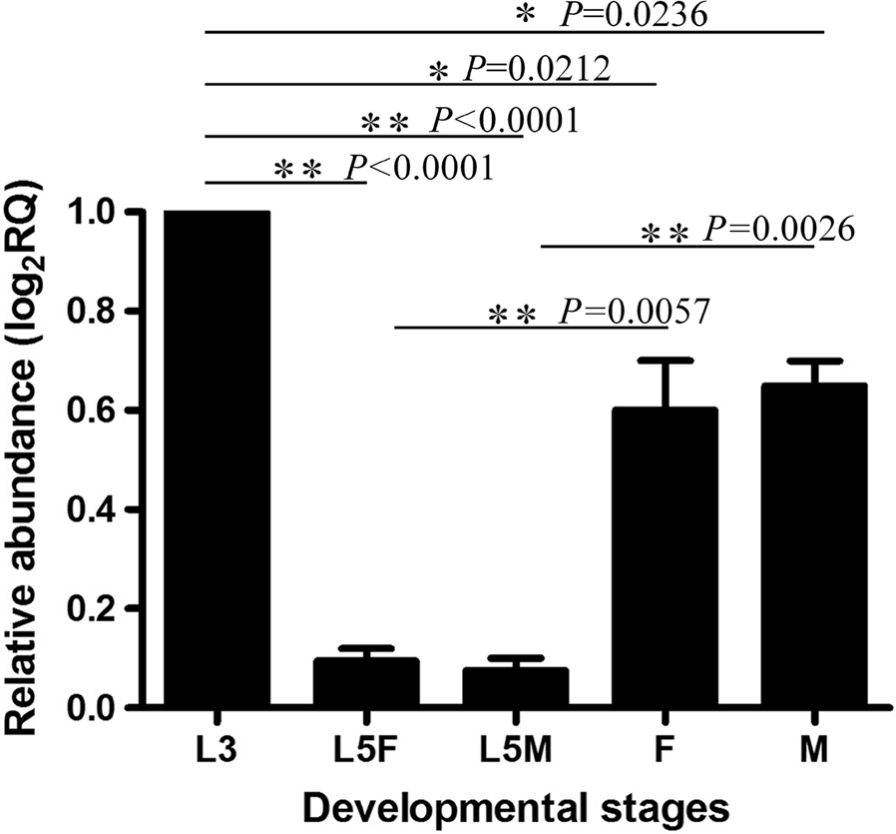
Transcriptional profile of *Acan-rps-30* in different developmental stages (third- and fifth-stage larvae [*L3*,* L5*, respectively], adult) and in different genders (females [*F*] and males [*M*]] of *A. cantonensis*, determined by real-time PCR analysis. Data shown are the mean ± standard error of the mean from three technical replicates with two biological replicates. Relative transcription of the *Acan-rps-*30 gene in each sample was calculated by normalization of the raw data, followed by the determination of abundance relative to the 18S ribosomal RNA gene (GenBank: AY295804), which served as an internal loading control. Statistical analysis was conducted using a one-way analysis of variance. Asterisks indicate statistical difference at *P < 0.05 and **P < 0.01

To determine the relative abundance of the *Acan*-*rps*-*30* transcript in different developmental stages (L3, L5 and adult) and genders (females [F] and males [M]) of the life-cycle of *A. cantonensis*, qRT-PCR was performed with the 18S ribosomal RNA gene as an internal loading control. The results showed that *Acan*-*rps*-*30* was transcribed in both the larval and adult developmental stages examined at different levels (Fig. 4; Additional file 2: Table S2). The expressions of *Acan*-*rps*-*30* were significantly downregulated in both *A. cantonensis* L5 and adults, compared with that in L3; furthermore, the expression level in L5 was much lower than that in the adult, possibly indicating the important roles of *Acan*-RPS-30 in different developmental stages (L3, L5 and adult) residing in different hosts.

**Fig. 5:**
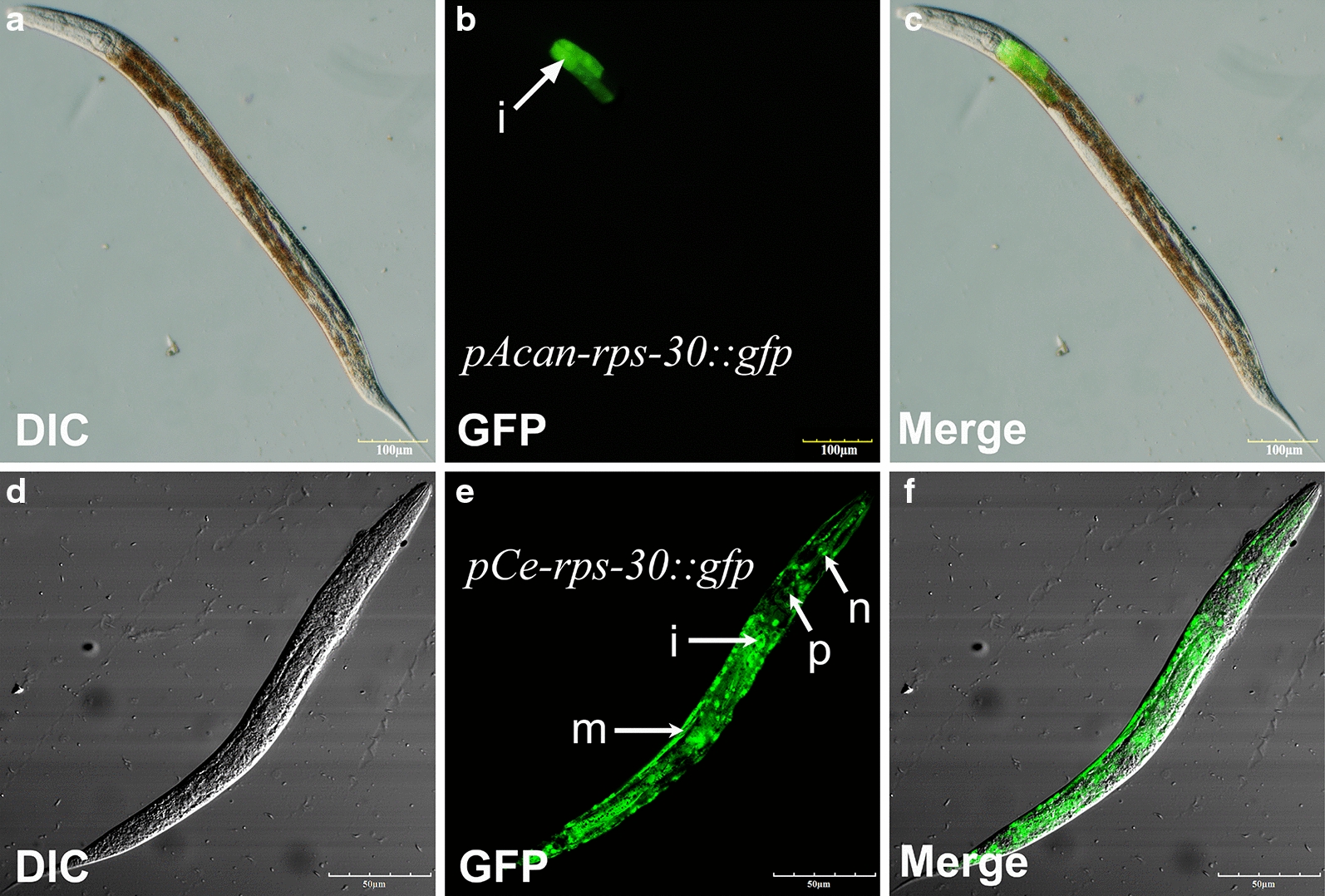
Expression pattern of the *A. cantonensis Acan-rps-*30 promoter in *Caenorhabditis elegans*. **a**–**c** The promoter activity of *Acan-rps-*30 in *C. elegans*. *pAcan-rps-*30*::gfp* is only expressed in cells in the intestine, mainly in the anterior end. **d**–**f** The promoter activity of *Ce-rps-*30. *pCe-rps-*30*::gfp* is expressed ubiquitously. Arrows indicate the different tissues studied: intestinal (*i*), muscle (*m*), neuron (*n*), pharynx (*p*).* DIC* Differential interference contrast microscopy

Due to the lack of functional genetic and* in vitro* culture methods, we were unable to detect the functions of *Acan*-RPS-30 directly in *A. cantonensis*. In the present study, we used *C. elegans*, proposed by numerous authors as a general model for many aspects of basic molecular, cellular and developmental biology in the less tractable parasitic nematodes [33–35], to investigate the anatomical expression patterns of *Acan*-*rps*-*30* in order to examine the closed evolutionary relationship between *A. cantonensis* and *C. elegans*, both of which belong to clade V according to cladistic analysis [33]. Wild-type *C. elegans* (N2 strain) were transformed with the construct *pAcan-rps-*30*::gfp* and *pCe-rps*30*::gfp*, respectively (Fig. 1a). Plasmid pRF4 was included in all transformations as a behavioral marker. Transgenic worms showing the roller phenotype were selected. The results showed that GFP under the promoter *pAcan-rps-*30 was only expressed in the intestine of *C. elegans*, mainly in the anterior end (Fig. 5a–c), which is the major tissue for lifespan regulation in *C. elegans* [36]. This is in contrast to the situation in the worms expressing *pCe-rps-*30*::gfp*, where GFP was expressed in almost all cells, including those of the intestine, nervous system, pharynx and muscle (Fig. 5d–f). The different activity of *pAcan-rps-*30 and *pCe-rps-*30 may be due to heterologous expression, with low promoter sequences similarity (data not shown). Therefore, *pCe-rps-*30 was used as the promoter in subsequent research on the functions of *Acan*-RPS-30 in *C. elegans*.

### Cross-species expressions of *Acan*-RPS-30 in *C. elegans* N2 strain and the *rps-30* deletion mutant worms

**Fig. 6:**
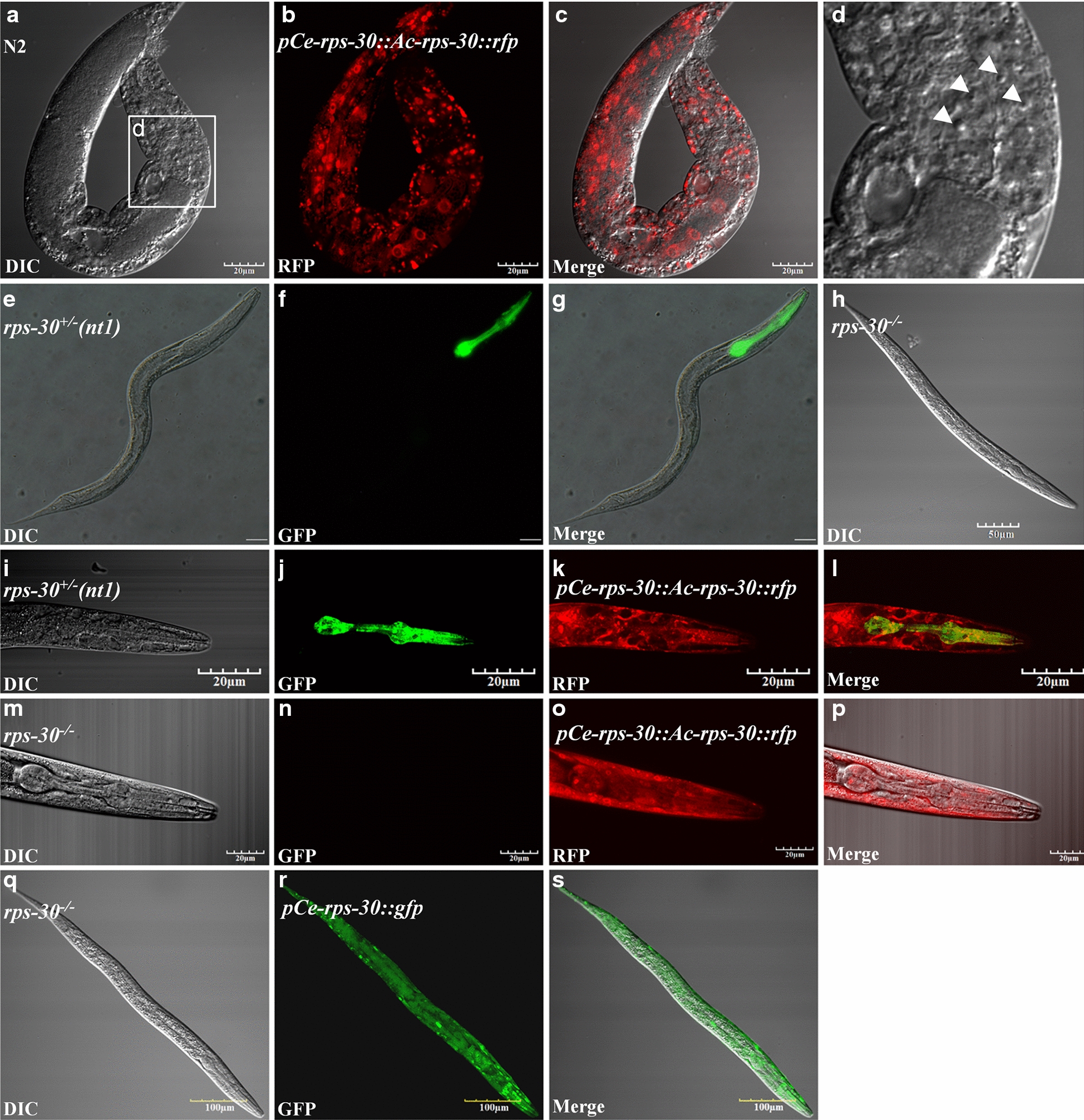
Cross-species expression of *Acan*-RPS-30 in *C. elegans* N2 strain and the *rps-*30 deletion mutant worms. **a**–**d** Expression of *pCe-rps-*30*::Acan-rps-*30*::rfp* in *C. elegans* N2 strain. RFP was expressed widely, but was RFP mainly focused on the nucleus; “button-like” apoptotic corpses were seen in the anterior pharynx. Arrowheads indicate apoptotic corpses. **e**–**g** The heterozygous *rps-*30^*+/–*^worm. The GFP fluorescence-positive worms (pharynx) carried the translocational balancer (nT1). **h** The homozygous *rps-*30^−/−^ worm. Worms without GFP (nT1) were mutation homozygous (*rps-*30^−/−^). **i**–**l** Expression of *pCe-rps-*30*::Acan-rps-*30*::rfp* in the heterozygous *rps-*30^+/−^ worm. RFP was expressed widely, and GFP fluorescence was positive in pharynx. **m**–**p** Expression of *pCe-rps-*30*::Acan-rps-*30*::rfp* in the homozygous *rps-*30^−/−^ worm. RFP was expressed widely, and GFP fluorescence was negative in pharynx. **q**–**s** Expression of *pCe-rps-*30*::gfp* in the homozygous *rps-*30^−/−^ worm

In order to clarify the role of *Acan*-RPS-30, cross-species expression of *Acan-rps-*30 in *C. elegans* was performed. The expressing constructs containing *Acan-rps-*30*::rfp* coding sequences driven by *Ce-rps-*30 promoters (Fig. 1b) were used to transform *C. elegans* N2 strain and *rps-*30 deletion mutant strain (*tm6034*), respectively. In N2 worms transformed with *pCe-rps*30*::Acan-rps-30::rfp*, RFP was expressed widely (Fig. 6b, c), consistent with the *pCe-rps*30*::gfp* expression pattern (Fig. 5d–f). In addition, RFP mainly focused on the nucleus for the existence of a NLS in the S30 region. “Button-like” apoptotic cell corpses arising from developmental apoptosis, which are the gold standard for quantification of apoptosis in *C. elegans* [25], were seen in the anterior pharynx (Fig. 6a, d), possibly suggesting the pro-apoptotic effect of *Acan*-RPS-30, consistent with the pro-apoptotic regulatory role of *Hs*-RPS-30 [19, 23].

In trans-heterozygous worms (*tm6034*), GFP fluorescence-positive animals (pharynx), carrying nT1 were heterozygous *rps-30*^+/−^ (Fig. 6e–g), and animals without GFP (nT1) were mutation homozygous *rps-30*^−/−^ (Fig. 6h). After the transformation of *pCe-rps*30*::Acan-rps-*30*::rfp* in *rps-30*^*+/–*^ worms, the offspring contained *rps-30*^*+/–*^ expressing *pCe-rps*30*::Acan-rps-*30*::rfp* (Fig. 6i–l) and *rps-*30^−/−^ expressing *pCe-rps30::Acan-rps-*30*::rfp* (Fig. 6m–p), with the *rps-*30^−/−^-expressing *pCe-rps*30*::gfp* (Fig. 6q–s) as the control in the following assay.

### Functional role of *Acan*-RPS-30 in oxidative stress

**Fig. 7:**
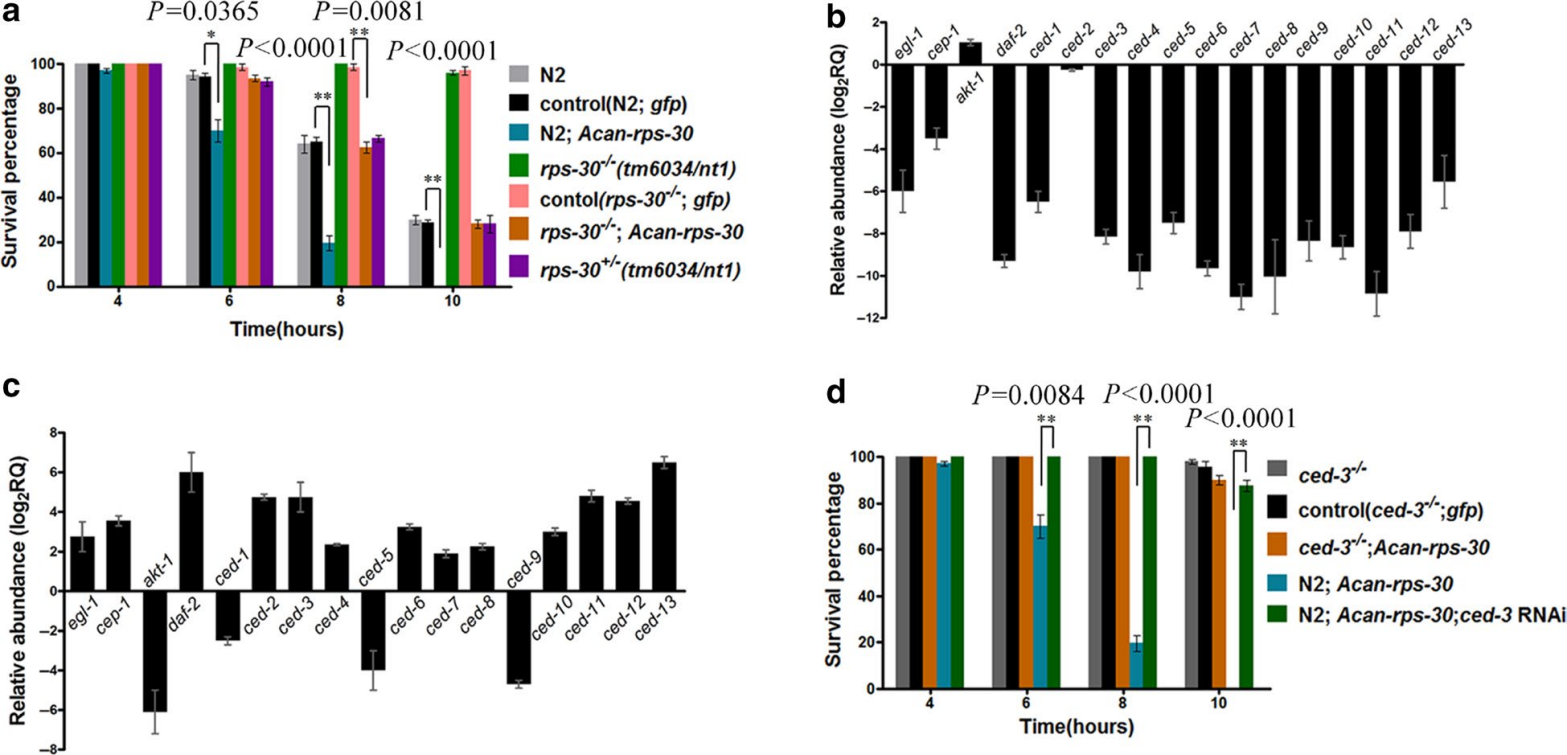
Downregulated RPS-30 plays a defensive role against oxidative stress by regulating *ced-*3. **a** Oxidative stress assays using H_2_O_2_ in* C. elegans* N2 strain and *rps-*30 mutant worms expressing *pCe-rps*30*::Acan-rps-*30*::rfp*. **b** The expression level of apoptosis genes in homozygous *rps-*30^−/−^ worm. **c** The expression level of apoptosis genes in* C. elegans* N2 worms expressing *pCe-rps*30*::Acan-rps-*30*::rfp*. **d** Oxidative stress assays using H_2_O_2_ in *ced-*3 mutant worms and worms expressing *pCe-rps*30*::Acan-rps-*30*::rfp*. The worms were counted as described in the Methods section. The error bars indicate standard deviation. Asterisks indicate signficant difference at *P < 0.05 and **P < 0.01

To investigate the role of *Acan*-RPS-30 in regulating oxidative stress resistance, we performed oxidative stress assays using H_2_O_2_. We found that the incidence of rapid death among the* C. elegans* N2 worms expressing *pCe-rps*30*::Acan-rps-*30*::rfp* was significantly higher than that among the N2 worms expressing *pCe-rps*30*::gfp*; in addition, the *rps-30* deletion mutants (*rps-*30^−/−^) were significantly more resistant than N2 worms. This oxidative stress resistance phenotype could be rescued and inhibited by expressing *pCe-rps30::Acan-rps-*30*::rfp* in *rps-*30^−/−^ mutant worms (Fig. 7a; Additional file 2: Table S2 ). These results may indicate the regulating role of *Acan*-RPS-30 in promoting susceptibility to oxidative stress.

As oxidative stress is considered to be one of the major factors that promote apoptosis [26], we next detected the expression levels of apoptosis genes in *C. elegans*. The results showed that all of the apoptosis genes were downregulated in *rps-*30^−/−^ mutant worms, with the exception of upregulation of *akt-1* (Fig. 7b), which inhibits CEP-1 and decreases DNA damage-induced apoptosis [37]. *ced-3* and *ced-4*, the core apoptosis executive genes [25], were both upregulated in N2 worms expressing *pCe-rps30::Acan-rps-30::rfp, *whereas *ced-9* was downregulated (Fig. 7c); this latter gene encodes the homologous protein to the anti-apoptotic B-cell lymphoma 2 (Bcl-2) family of proteins [38]. These results may indicate the role of *Acan*-RPS-30 in promoting apoptosis in *C. elegans*.

To further determine the effect of apoptosis regulated by *Acan-*RPS-30 on oxidative stress susceptibility, we constructed the *C. elegans* strain *ced-*3^−/−^ (*ok2734*) expressing *pCe-rps*30:*:Acan-rps-*30*::rfp* and the strain N2 expressing *pCe-rps*30*::Acan-rps-*30*::rfp* with *ced-3* knockdown using RNAi. The survival percentages were detected with the strains *ced-*3^−/−^ expressing *pCe-rps30::gfp* and strain N2 expressing *pCe-rps*30*::Acan-rps-*30*::rfp* as controls, respectively. We found that the incidence of rapid death among the *ced-*3^−/−^ worms expressing *pCe-rps*30*::Acan-rps-*30*::rfp* was almost the same as that among the *ced-*3^−/−^ worms expressing *pCe-rps*30*::gfp*; and that the incidence of rapid death among the N2 worms expressing *pCe-rps*30*::Acan-rps-*30*::rfp* was significantly higher than that among the N2 worms expressing *pCe-rps*30*::Acan-rps-*30*::rfp* with *ced-*3 knocked down (Fig. 7d; Additional file 2: Table S2). These results may suggest that the regulating role of *Acan-*RPS-30 in promoting susceptibility to oxidative stress plays through CED-3, which is the core executive effector in worm cell apoptosis [25].

## Discussion

Eosinophilic meningitis, caused by *A. cantonensis* L5, is mainly attributed to the eosinophils [39], which contribute to tissue inflammatory responses in helminthic infections [8]. Eosinophil are well-equipped immune cells recruited from the circulation into inflammatory foci [40] that directly recognize helminth-derived immunomodulating agents and function in host defense mechanisms against helminth infection [8]. The cell surface of eosinophils possess a variety of receptors for cell signaling associated with chemotaxis, adhesion, respiratory burst, degranulation, apoptosis or survival [41], all of which may be closely associated with eosinphil-mediated tissue inflammatory responses in helminth infection [8]. Eosinophils primarily contain four main granules: crystalloid granules, primary granules, small granules and secretory vesicles [42]. Cytotoxic granular proteins, including the major basic proteins, EPO, eosinophil cationic protein and eosinophil-derived neurotoxin, reside in the crystalloid granules [9, 10]. The functional role of EPO is associated with the killing of helminths killing [43]. EPO catalyzes the peroxidative oxidation of halides and thiocyanate present in the plasma together with H_2_O_2_ generated by dismutation of the superoxide produced during respiratory burst [11–13]. Eosinophils, the robust producers of extracellular superoxide due to the expression of high levels of the enzyme complex that generates superoxide [7], produce superoxide anions in response to helminth-derived cysteine proteases [44]. However, helminthic worms residing in the host with high level of eosinophils have evolved to attenuate eosinophil-mediated tissue inflammatory responses for their survival in hosts, such as inducing the apoptosis of eosinophils [45, 46] and blocking the chemotactic effects on eosinophils [47]. In this study, we identified *Acan-rps-30* from *A. cantonensis*. The expression of *Acan*-*rps-30* was significantly downregulated in both L5 and adult *A. cantonensis*. It is known that both L5 and adult *A. cantonensis* residing in mammalian, humans and rats, respectively, are attacked by the immune response from hosts, such as superoxide produced by eosinophils. Our results show that *Acan-*RPS-30 could promote susceptibility to H_2_O_2_ and that *rps-*30^−/−^ mutant worms were resistant to oxidative stress. This observation might indicate the regulating function of *Acan-*RPS-30 in attenuating eosinophil-mediated immue attack upon L5 worms in the central nervous system of humans by due to a lower expression. In comparison L3 worms, with a higher level of *Acan*-*rps-*30, reside in intermediate hosts (e.g. *Pomacea canaliculata*) in which the immune system is lower than that in mammalians, or the immune attack may be weaker, or even there may be no eosinophil-mediated superoxide attack. Therefore, the higher level of *Acan*-*rps-30* in L3 worms may indicate its multi-function in different developmental stages, such as promoting the development of L3 worms with the S30 region [22]. Furthermore, the expression level in L5 was significantly lower than that in adult, which possesses a thicker cuticle than L5 larva. Alhough adult worms in the blood vessels of the hearts and lungs of rats, in which there is an active immune system, are attacked by superoxide from eosinophils, the thick cuticle may provide some protection [48]. In addition, many other proteins may be differently expressed in the cuticle, such as the homologous gene of *lec-1*, which plays an important role against damage due to oxidative stress [4, 48].

*Angiostrong cantonensis* is relatively closely related to the model organism *C. elegans*, with both belonging to clade V [2, 33], and the homologous gene of *Acan-rps-30* is *Ce-rps30* (C26F1.4). Here, we used *C. elegans* as a surrogate to explore the* in vivo* functions of the homologous gene *Acan-rps-30* for the lack of effective genetic manipulation in parasitic nematode. In *C. elegans*, apoptosis is characterized by the refractile “button-like” apoptotic corpses that are the result of inefficient engulfment from healthy neighboring cells [49–51]. The “button-like” appearance under differential interference contrast (DIC) optics is the gold standard for quantification of apoptosis in *C. elegans* [25]. In this study, the “button-like” corpses were seen in the anterior pharynx of the transgenic worm expressing *pCe-rps*30*::Acan-rps-*30*::rfp*, indicating that apoptosis was occurring. CED-1 and CED-5 proteins can recognize corpses and are critical to engulfment [49]. The downregulated expression of *ced-1* and *ced-*5 in the transgenic worm expressing *pCe-rps*30*::Acan-rps-*30*::rfp* may contribute to the formation of corpses.

Four genes, comprising the core apoptosis pathway in *C. elegans*, have been identified [37, 52]. *egl-1* encodes a proapoptotic BH3-only protein that antagonizes the CED-9 protein [53]. *ced-*9, which functions upstream of *ced-*4 to prevent activation of the CED-3 caspase, encodes the homologous protein to the anti-apoptotic B-cell lymphoma 2 (Bcl-2) family of proteins [38]. *ced-*3 encodes a proteolytic caspase protein that is activated by CED-4, the worm homologue of mammalian apoptotic protease activation factor 1 [54]. Therefore, CED-3 is the core executioner [25]. In the worms expressing *pCe-rps*30*::Acan-rps-*30*::rfp*, the *ced-3* was upregulated and the worms exhibited apoptosis and susceptibility to oxidative stress; whereas in the *rps-*30^−/−^ mutant worms, the *ced-*3 was downregulated and the worms exhibited resistance to oxidative stress. This phenotype could be converted with the *ced-*3 defective mutation and RNAi. Therefore, the function of *Acan-*RPS-30 in promoting susceptibility to oxidative stress may possibly be conducted through apoptosis by regulating CED-3. In *A. cantonensis* L5, *Acan-*RPS-30 was downregulated to enhance the resistance to oxidative stress from eosinophils to ensure wormssurvival in host.

## Conclusions

This study investigated the structural and functional characterization of *Acan*-RPS-30 from *A. cantonensis*. We found that *Acan*-RPS-30 could promote worms to be susceptible to oxidative stress through apoptosis by regulating CED-3 and that worms with *Acan*-RPS-30 downregulated were resistant to oxidative stress. Our findings may reveal the mechanism for *A. cantonensis* L5 worms surviving in the central nervous system of humans from immune attack by eosinophils.

## Supplementary information

**Additional file 1: Table S1.** List of primers used in this study.

**Additional file 2: Table S2.** Statistical comparisons of data presented in figures.

## Data Availability

Data supporting the conclusions of this article are included within the article and its additional files. The datasets used in the present study are available from the corresponding author upon reasonable request.

## Abbreviations

*Acan-rps-*30: Homologous gene of human* Fau*
AKT: Protein kinase B
CED: Cell death abnormality
CEP-1: *Caenorhabditis elegans* P-53-like protein
EGL: Egg-laying defective
FBR-MuSV: Finkel-Biskis-Reilly murine sarcoma virus
Fox: FBR osteosarcoma X
GFP: Green fluorescent protein
RNAi: RNA interference
RPF: Red fluorescent protein
