# Supplementary material for: Downregulated RPS-30 in Angiostrongylus cantonensis L5 plays a defensive role against damage due to oxidative stress
Source: Parasit Vectors. 2020 Dec 9;13:617. doi: 10.1186/s13071-020-04495-3 (PMC7724845; doi:10.1186/s13071-020-04495-3)
Supplement: Supplementary file 1 — Additional file 1: Table S1. List of primers used in this study. [file 13071_2020_4495_MOESM1_ESM.doc]

**Additional file 1: Table S1. List of primers used in this study.**

| Primer name | | Gene amplified | Used for | | Sequence (5`-3`) | |
| --- | --- | --- | --- | --- | --- | --- |
| rps-30DF | | Ac-rps-30 | PCR | | CAAGTNTAYGARTGGATGGT | |
| rps-30DR | | Ac-rps-30 | PCR | | CCNGCNCCYTCRTTYTG | |
| GW-Ac-rps-30-F1 | | Ac-rps-303`flanking | Genome walking | | GCTTCCGCTCACGTATCAGA | |
| GW-A c-rps-30-F2 | | Ac-rps-30 3`flanking | Genome walking | | AGTCGCGCAGGCAAATTCCG | |
| GW-Ac-rps-30-F3 | | Ac-rps-30 3`flanking | Genome walking | | CTTTCGCAAACAACGGATCA | |
| GW-Ac-rps-30-R1 | | Ac-rps-305`flanking | Genome walking | | CTTGCTGGTGGTGTCGAGGG | |
| GW-Ac-rps-30-R2 | | Ac-rps-30 5`flanking | Genome walking | | CGTCGGGGATTTCGGCCC | |
| GW-Ac-rps-30-R3 | | Ac-rps-30 5`flanking | Genome walking | | AGATTATGTCGAATTGAGTT | |
| GW-Ac-rps-30-R4 | | Ac-rps-30 5`flanking | Genome walking | | CTGCATTCATGGTGCTCAT | |
| GW-Ac-rps-30-R5 | | Ac-rps-30 5`flanking | Genome walking | | TAGTCGCGTTCGCCGGATAACG | |
| GW-Ac-rps-30-R6 | | Ac-rps-30 5`flanking | Genome walking | | AGGACACACTTGTGGCAGCAA | |
| RACE-3`F1 | | Ac-rps-30 cDNA | RACE | | CATAATCTGTCGCTGCACTCG | |
| RACE-3`F2 | | Ac-rps-30 cDNA | RACE | | GGTGGGTGATCAATCCGGA | |
| RACE-5`R1 | | Ac-rps-30 cDNA | RACE | | CGTCGTGTCGTGGATTTCTTG | |
| RACE-5`R2 | | Ac-rps-30 cDNA | RACE | | GATCGGCATAGCTGAGATTTC | |
| pA c-rps-30-F | | *p* Ac-rps-30 | expression patterns | | CCCAAGCTTCGTTAGAAGCTTGTCTAGCT | |
| pA c-rps-30-R | | *p* Ac-rps-30 | expression patterns | | TCCCCCGGGCGCTGCTCGACGACGGTGGC | |
| pCe-rps-30-F | | *p* Ce-rps-30 | expression patterns | | CCCAAGCTTCCTTTGTGCACTGGCGAATTC | |
| p Ce-rps-30-R | | *p* Ce-rps-30 | expression patterns | | TCCCCCGGGACTAGATGGACGAGGTGAA | |
| Ac-rps-30-F | | Ac-rps-30 | expression | | TGCTCTAGAATGAGCACCATGAATGCAGC | |
| Ac-rps-30-R | | Ac-rps-30 | expression | | TGCTCTAGAGAGATTGTCAAAATTGAGGC | |
| real-egl-1-F | | egl-1 | RT-PCR | | AGCACCATGAATGCAGCCAA | |
| real-egl-1-R | | egl-1 | RT-PCR | | GCTCATGTATCAACGGACTC | |
| real-cep-1-F | | cep-1 | RT-PCR | | GCGTCGGTCTCGTCGTCTTT | |
| real-cep-1-R | | cep-1 | RT-PCR | | TCGCTCGGCTTCTTACGACA | |
| real- Ac-18S-F | | *18srna* | RT-PCR | | TGGATCTGAGTTGCATGCA | |
| real- Ac-18S-R | | *18srna* | RT-PCR | | CGCGCAGGGATACGAATGC | |
| real-daf-2-F | *daf-2* | | | RT-PCR | | TGCCGAAAATGCGTTGGCAAGT |
| real-daf-2-R | *daf-2* | | | RT-PCR | | CCGGAACATGTTCAACGAGAT |
| real-AKT-1-F | *akt-1* | | | RT-PCR | | AGGGATGGCTTCACAAGAAAG |
| real-AKT-1-R | *akt-1* | | | RT-PCR | | CGCATAGAATGTTCGCTCAATG |
| real-ced-1-F | *ced-1* | | | RT-PCR | | TGAGCCGGGAAAATGTGAATG |
| real-ced-1-R | *ced-1* | | | RT-PCR | | CATCGTTCTCCTTGAAATCCAC |
| real-ced-2-F | *ced-2* | | | RT-PCR | | AATCGCGAATCAGTCGTTTCC |
| real-ced-2-R | *ced-2* | | | RT-PCR | | CACCAATCCTGGTTCGTTTTTG |
| real-ced-3-F | *ced-3* | | | RT-PCR | | CGACGAGAAAACCATGTACAGA |
| real-ced-3-R | *ced-3* | | | RT-PCR | | GGCAAAGTCTCGAATTGTCAG |
| real-ced-4-F | *ced-4* | | | RT-PCR | | CGCAATGGCTCTTCAAAGATG |
| real-ced-4-R | *ced-4* | | | RT-PCR | | GCATTCGTTTTCCACTGAGAAG |
| real-ced-5-F | *ced-5* | | | RT-PCR | | CGTGGAATGTGTGGATAGTCTT |
| real-ced-5-R | *ced-5* | | | RT-PCR | | GCATTGTGGCATGTCGAAGTA |
| real-ced-6-F | *ced-6* | | | RT-PCR | | GGATTCATCCGCCAGATTATC |
| real-ced-6-R | *ced-6* | | | RT-PCR | | AGTGGAAATTCGCATCTCGATC |
| real-ced-7-F | *ced-7* | | | RT-PCR | | GTGAAGAACAACTGCTGACTG |
| real-ced-7-R | *ced-7* | | | RT-PCR | | CTTGGTCCATACGGATTGTAAC |
| real-ced-8-F | *ced-8* | | | RT-PCR | | GCTGAAAGGGATGCAACATTG |
| real-ced-8-R | *ced-8* | | | RT-PCR | | GTAACGAGCGGTTTTGAACAAC |
| real-ced-9-F | *ced-9* | | | RT-PCR | | CACGCGGAAAATTTTGAGACC |
| real-ced-9-R | *ced-9* | | | RT-PCR | | CCACGGATTCCATCATTTTTGC |
| real-ced-10-F | *ced-10* | | | RT-PCR | | ATGCAAGCGATCAAATGTGTCG |
| real-ced-10-R | *ced-10* | | | RT-PCR | | CGATCGTAATCTTCCTGTCCA |
| real-ced-11-F | *ced-11* | | | RT-PCR | | TTCCATCCGAACCAATGAGTC |
| real-ced-11-R | *ced-11* | | | RT-PCR | | TGGCACATAATTCACAGCTGTC |
| real-ced-12-F | *ced-12* | | | RT-PCR | | TGTGCATCACCGGATCATTATG |
| real-ced-12-R | *ced-12* | | | RT-PCR | | TACAGCTAATTCCAGCATACTTG |
| real-ced-13-F | *ced-13* | | | RT-PCR | | TGATGTCGTACAAGCGTGATG |
| real-ced-13-R | *ced-13* | | | RT-PCR | | TCAAACTCGTCGCACATAACTG |
| RNAi-ced-3-F | | *ced-3* | RNAi cloning | | AGTACACAGAGTGTGATAATGC | |
| RNAi-ced-3-R | | *ced-3* | RNAi cloning | | GCAAACTCCATCACAAAACAG | |

RT-PCR, real-time PCR; RACE, rapid amplification of cDNA ends; RNAi, RNA interference.
