# Supplementary material for: Downregulated RPS-30 in Angiostrongylus cantonensis L5 plays a defensive role against damage due to oxidative stress
Source: Parasit Vectors. 2020 Dec 9;13:617. doi: 10.1186/s13071-020-04495-3 (PMC7724845; doi:10.1186/s13071-020-04495-3)
Supplement: Supplementary file 2 — Additional file 2: Table S2. Statistical comparisons of data presented in figures. [file 13071_2020_4495_MOESM2_ESM.docx]

**Additional file 2: Table S2.** Statistical comparisons presented in the figures

**Figure 4**

**ANOVA: F(4,10)=56.428, p<0.001**

| Comparing groups | P value corrected by LSD |
| --- | --- |
| L3 vs L5F | <0.0001 |
| L3 vs L5M | <0.0001 |
| L3 vs F | 0.0212 |
| L3 vs M | 0.0236 |
| L5F vs F | 0.0057 |
| L5M vs M | 0.0026 |

**Figure 7a**

| Time (h) | Comparing groups | *t*-test statistic and P-value |
| --- | --- | --- |
| 6 | Control (N2; *gfp*) vs N2; *Acan-rps-30* | t(4) =3.423, P = 0.0365 |
| 8 | Control (N2; *gfp*) vs N2; *Acan-rps-30* | t(4) =20.294, P <0.0001 |
|  | Control (*rps-30^–/–^*; *gfp*) vs *rps-30^–/–^*; *Acan-rps-30* | t(4) =4.903, P=0.0081 |
| 10 | Control (N2; *gfp*) vs N2; *Acan-rps-30* | t(4) =38.478, P < 0.0001 |

**Figure 7d**

| Time (h) | Comparing groups | *t*-test statistic and P-value |
| --- | --- | --- |
| 6 | N2; *Acan-rps-30* vs N2; *Acan-rps-30*; *ced-3* RNAi | t(4) = -5.002, P = 0.0084 |
| 8 | N2; *Acan-rps-30* vs N2; *Acan-rps-30*; *ced-3* RNAi | t(4) = -35.467, P <0.0001 |
| 10 | N2; *Acan-rps-30* vs N2; *Acan-rps-30*; *ced-3* RNAi | t(4) = -50.397, P < 0.0001 |
